# Elevated X-linked inhibitor of apoptosis protein (XIAP) expression uncovers detrimental prognosis in subgroups of neoadjuvant treated and T-cell rich esophageal adenocarcinoma

**DOI:** 10.1186/s12885-019-5722-1

**Published:** 2019-05-31

**Authors:** Lars M. Schiffmann, Heike Göbel, Heike Löser, Fabian Schorn, Jan Paul Werthenbach, Hans F. Fuchs, Patrick S. Plum, Marc Bludau, Thomas Zander, Wolfgang Schröder, Christiane J. Bruns, Hamid Kashkar, Alexander Quaas, Florian Gebauer

**Affiliations:** 1 Cologne Excellence Cluster on Cellular Stress Responses in Aging-Associated Diseases (CECAD), Center for Molecular Medicine Cologne (CMMC) and Institute for Medical Microbiology, Immunology and Hygiene, University of Cologne, CECAD Research Center, Joseph-Stelzmann-Str. 26, 50931 Cologne, Germany; 2 Department of General, Visceral and Cancer Surgery, University of Cologne, Kerpener Str. 62, 50937 Cologne, Germany; 3 Center for Integrated Oncology (CIO) Cologne Bonn, Kerpener Str. 62, 50924 Cologne, Germany; 4 Institute of Pathology, University of Cologne, Kerpener Str. 62, 50937 Cologne, Germany; 5 Department I of Internal Medicine, University of Cologne, Kerpener Str. 62, 50924 Cologne, Germany

**Keywords:** XIAP, EAC, Biomarker of response, Outcome prediction

## Abstract

**Background:**

Molecular markers predicting survival in esophageal adenocarcinoma (EAC) are rare. Specifically, in favorable oncologic situations, e.g. nodal negativity or major neoadjuvant therapy response, there is a lack of additional risk factors that serve to predict patients’ outcome more precisely. This study evaluated X-linked inhibitor of apoptosis protein (XIAP) as a potential marker improving outcome prediction.

**Methods:**

Tissue microarrays from 362 patients that were diagnosed with resectable EAC were included in the study. XIAP was stained by immunohistochemistry and correlated to clinical outcome, molecular markers and markers of the cellular tumor microenvironment.

**Results:**

XIAP did not impact on overall survival (OS) in the whole study collective. Subgroup analyses stratifying for common genetic markers (TP53, ERBB2, ARID1A/SWI/SNF) did not disclose any impact of XIAP expression on survival. Detailed subgroup analyses of [1] nodal negative patients, [2] highly T-cell infiltrated tumors and [3] therapy responders to neoadjuvant treatment revealed a significant inverse role of high XIAP expression in these specific oncologic situations; elevated XIAP expression detrimentally affected patients’ outcome in these subgroups. [1]: OS XIAP low: 202 months (m) vs. XIAP high: 38 m; [2]: OS 116 m vs. 28.2 m; [3]: OS 31 m vs. 4 m).

**Conclusions:**

Our data suggest XIAP expression in EAC as a worthy tool to improve outcome prediction in specific oncologic settings that might directly impact on clinical diagnosis and treatment of EAC in the future.

**Electronic supplementary material:**

The online version of this article (10.1186/s12885-019-5722-1) contains supplementary material, which is available to authorized users.

## Background

Esophageal adenocarcinoma (EAC) shows an increasing incidence over the last decades in the western world [1]. Multi-disciplinary treatment strategies including intense neoadjuvant treatment regimens and radical oncologic surgery continually contribute to improved survival rates. Though overall prognosis is limited and EAC ranks on 6th place for cancer associated death [2]. Most patients are diagnosed with advanced tumor stages including presence of lymph node metastasis and locally advanced tumor stages. Since the tumor infiltration depth (pT) and presence of lymph node metastasis are the main pathological factors predicting long-term survival in EAC, within particular pathological subgroups, the overall-survival differs significantly without prior knowledge of the individual patients’ prognosis. For this reason, there is a mandatory need for the identification of biomarkers allowing the stratification of patients with similar pT and pN stages into high- and low-risk patients.

The X-linked inhibitor of apoptosis protein (XIAP) has been frequently shown to be upregulated in different cancer entities [3–6]. Besides its anti-apoptotic function [7] XIAP was additionally shown to promote cellular inflammatory signaling and trigger cytokine secretion [8–10].

The objective of this study is to assess the significance of XIAP expression as a predictor of overall survival in EAC. We analyzed XIAP in the largest collective of EAC so far. Our data show for the first time that in different generally favorable clinical situations (tumor response to neoadjuvant therapy, in nodal negativity and in highly T-cell infiltrated tumors) XIAP expression can be used to identify patients that have a poor prognosis which is not predictable with current state of the art staging methods.

## Methods

### Patients and tumor samples

Formalin-fixed and paraffin embedded material of 362 patients with esophageal adenocarcinomas that underwent primary surgical resection or resection after neoadjuvant therapy between 1999 and 2014 at the Department of General, Visceral and Cancer Surgery, University of Cologne, Germany were analyzed. Standard surgical procedures were either transthoracic esophagectomy with lymphadenectomy of the mediastinal and abdominal compartment (2-field LAD), transhiatal esophagectomy with lymphadenectomy of the lower mediastinum or transhiatal extended gastrectomy with D2-lymphadenectomy.

Patients with locally advanced esophageal cancer (cT3) or evidence for locoregional lymph node metastasis in clinical staging received preoperative chemoradiation or chemotherapy according to established protocols within national guidelines [11–14].

Construction of the tissue-micro arrays (TMA) was performed as previously described [15, 16]. In brief, tissue cylinders with a diameter of 1.2 mm each were punched from selected tumor tissue blocks using a self-constructed semi-automated precision instrument and embedded in empty recipient paraffin blocks. 4 μm sections of the resulting TMA blocks were transferred to an adhesive coated slide system (Instrumedics Inc., Hackensack, NJ) for immunohistochemistry (IHC).

All procedures performed in studies involving human participants were in accordance with the ethical standards of the institutional research committee and with the 1964 Helsinki declaration and its later amendments or comparable ethical standards.

### Immunohistochemistry (IHC)

IHC was performed on TMA slides. Tumor cell XIAP was detected using a polyclonal rabbit anti-XIAP antibody (ab21278: dilution 1:1000) on Leica BOND-MAX stainer (Leica Biosystems, Germany) according to the manufacturers’ protocol.

We correlated the XIAP results with previously collected and described data like T-cell inflammation of the tumor microenvironment and different molecular tumor cell alterations like TP53, ARIDa1 loss and ERBB2- amplification [17, 18].

### Analysis

The evaluation of immunohistochemical expression scores was peformed manually by high-level trained pathologists (HG, HL) independently and in a blinded fashion to any clinical details. The following scores were used for the analysis:

XIAP: no staining was considered as negative (0), a weak staining intensity in less than 70% or a moderate staining intensity in less than 30% of tumor cells was considered as weak [1], a moderate staining intensity in less than 70% or a weak staining intensity in more than 70% or a high tumor intensity in less than 30% of tumor cells as moderate [2] and a high staining intensity in more than 30% of the tumor cells or moderate intensity in more than 70% was considered as high [3].

### Statistical analysis

Clinical data were collected prospectively according to a standardized protocol. SPSS Statistics for Mac (Version 24, IBM) was used for statistical analysis. Interdependence between stainings and clinical data were calculated using Fisher’s exact tests. Survival curves were plotted using the Kaplan-Meier method and analyzed using the log-rank test. All tests were two-sided. *P* values < 0.05 were considered statistically significant.

## Results

TMA spots from 362 resected EACs (UICC stages I-III) were stained for XIAP. 311 tumor-containing spots (85.9%) were eligible to be scored for XIAP expression by a pathologist blinded to any clinical details. Reasons for non-informative cases (51 spots; 14.1%) included lack of tissue samples or absence of unequivocal cancer tissue in the TMA spot. XIAP was determined negative in 28 cases (7.7%). Score 1 was detected in 132 patients (36.5%), score 2 in 107 (29.6%) and score 3 in 44 patients (12.2%). For further analyses, patients were stratified for score 0–2 which was summarized as XIAP low (*n* = 267, 85.9%) and score 3 which was considered to be XIAP high (*n* = 44, 14.1%). Public domain data analyses revealed high mRNA levels for XIAP in 9.2% of the cases for EAC (data not shown) which validates our findings [19, 20]. Representative images of XIAP low and XIAP high tumors are depicted in Additional file 4: Figure S4. Table 1 depicts clinical characteristics of these 311 XIAP low or high patients included in the study.

**Table 1:** Basic clinical and demographic characteristics of studied patients.

	total		XIAP low		XIAP high		p
	n	%	n	%	n	%	
number of patients	311	100	267	85.9	44	14.1	
sex							
female	32	10.3	29	90.6	3	9.4	0.593
male	279	89.7	238	85.3	41	14.7	
age at diagnosis			62.1		66.3		0.032
initial T stage (^a^)							
pT1	25	8.1	21	84	4	16	0.151
pT2	29	9.4	29	100	0	0	
pT3	250	80.6	211	84.4	39	15.6	
pT4	6	1.9	5	83.3	1	16.7	
initial *N* stage (^a^)							
pN0	126	40.6	104	82.5	22	17.5	0.260
pN1	113	36.5	101	89.4	12	10.6	
pN2	36	11.6	29	80.6	7	19.4	
pN3	35	11.3	32	91.4	3	8.6	
Grading							
G1	3	1.4	3	100	0	0	0.539
G2	136	62.4	112	82.4	24	17.6	
G3	78	35.8	69	88.5	9	11.5	
G4	1	0.5	1	100	0	0	
R status (^c^)							
R0	206	66.2	175	85	31	15	0.424
R1	16	5.1	14	87.5	2	12.5	
R2	2	0.6	1	50	1	50	
neoadjuvant therapy (^b^)							
yes	185	60.3	165	89.2	20	10.8	0.064
no	122	39.7	99	81.1	23	18.9	
type of neoadj. Therapy (‘)							
Rctx	107	93	86	80.4	21	19.6	0.620
Ctx	8	7	7	87.5	1	12.5	

^a^, ^b^,^c^,’ indicate missing clinical information for the respective category (^a^: *n* = 1 missing, ^b^: *n* = 4 missing, ^c^ *n* = 87 missing, ‘=74 missing)

**Fig. 1:**
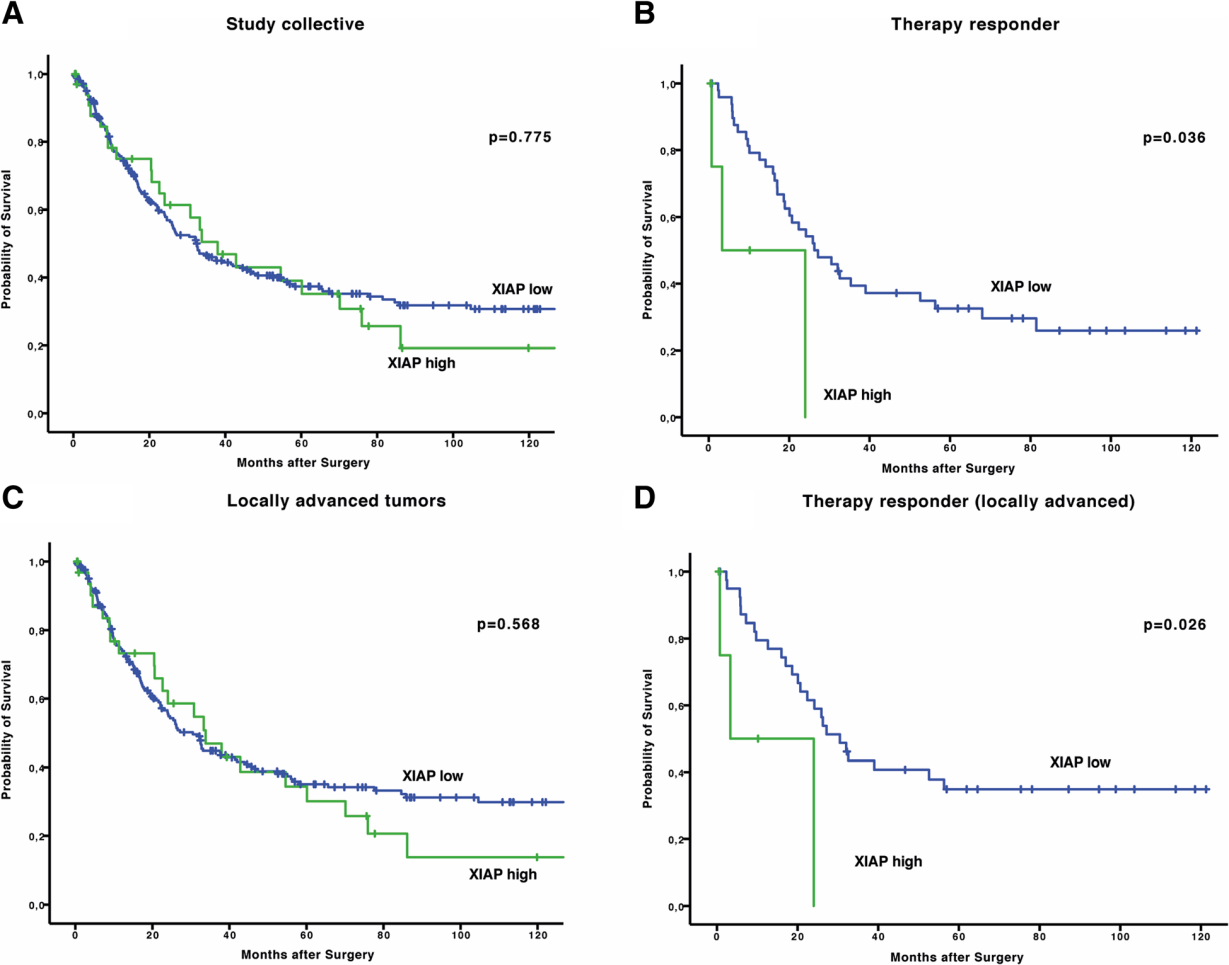
**a** Kaplan-Meier curve showing OS of patients with EAC in dependence on XIAP status in the total collective **b** Kaplan-Meier curve showing OS of patients that responded to neoadjuvant therapy with EAC in dependence on XIAP status **c** Survival curves depicting OS of patients in locally advanced tumor stages (pT3, T4,Nx) **d** Kaplan-Meier curve showing OS of therapy responders depending on XIAP status

In XIAP low versus XIAP high expressing tumors the overall-survival (OS) did not significantly differ in the complete study collective. Median OS was 32.5 months (95% CI 24.9–40.0 months) in the XIAP low compared to 38.0 months (95% CI 23.2–52.7 months, *p* = 0.775) in the XIAP high group (Fig. 1).

As XIAP is thought to be involved in therapy resistance [5] due to decreased cell death in XIAP overexpressing tumors we investigated whether XIAP levels can be correlated to treatment response (to combined radio- chemotherapy or chemotherapy alone in a neoadjuvant fashion) measured by a histopathological regression score. Regression scores were available from 133 patients of the eligible patients. Histopathological regression score ranged from 1 to 3; 1 was considered as non-responder, 2 and 3 as responders (modified from [21]). Histopathological non-responders did not have a higher XIAP score per se indicating that XIAP in EAC does not result in increased resistance to neoadjuvant therapy (Table 2).

**Table 2:** Cross-table showing distribution of therapy responders stratified for XIAP low vs. XIAP high expression

		low	high	Total	p
Response	no	65	11	76	
	yes	51	6	57	0.604
	Total	116	17	133	

When we plotted survival stratified for therapy response we saw that XIAP expression in therapy responders is capable of identifying patients that have a worse overall prognosis though they responded to therapy. Median OS in XIAP low, therapy responsive patients was 26.3 months (95% CI 0.0–18.5 months) compared to 3.3 months (95% CI 0.0–26.2 months, *p* = 0.036, Fig. 1b).

This led us to the hypothesis that XIAP might be more important in general in neoadjuvant treated patients than in chemo and/or radiation naïve patients. According to national and international guidelines neoadjuvant therapy is conducted in locally advanced stages (cT3, cT4). We therefore performed subgroup analysis for T3 and T4 patients. In general, also in this group, XIAP expression did not affect overall survival (XIAP low: median OS 30.5 months (95% CI 24.3–36.75 months) vs. XIAP high: 33.8 months (95% CI 17.1–50.5 months; *p* = 0.568)) but was able to predict poor outcome in therapy responders (XIAP low: median OS 30.5 months (95% CI 22.4–38.6 months) vs. XIAP high: 3.3 months (95% CI 0.0–28.5 months; *p* = 0.026)), (Fig. 1c, d).

**Fig. 2:**
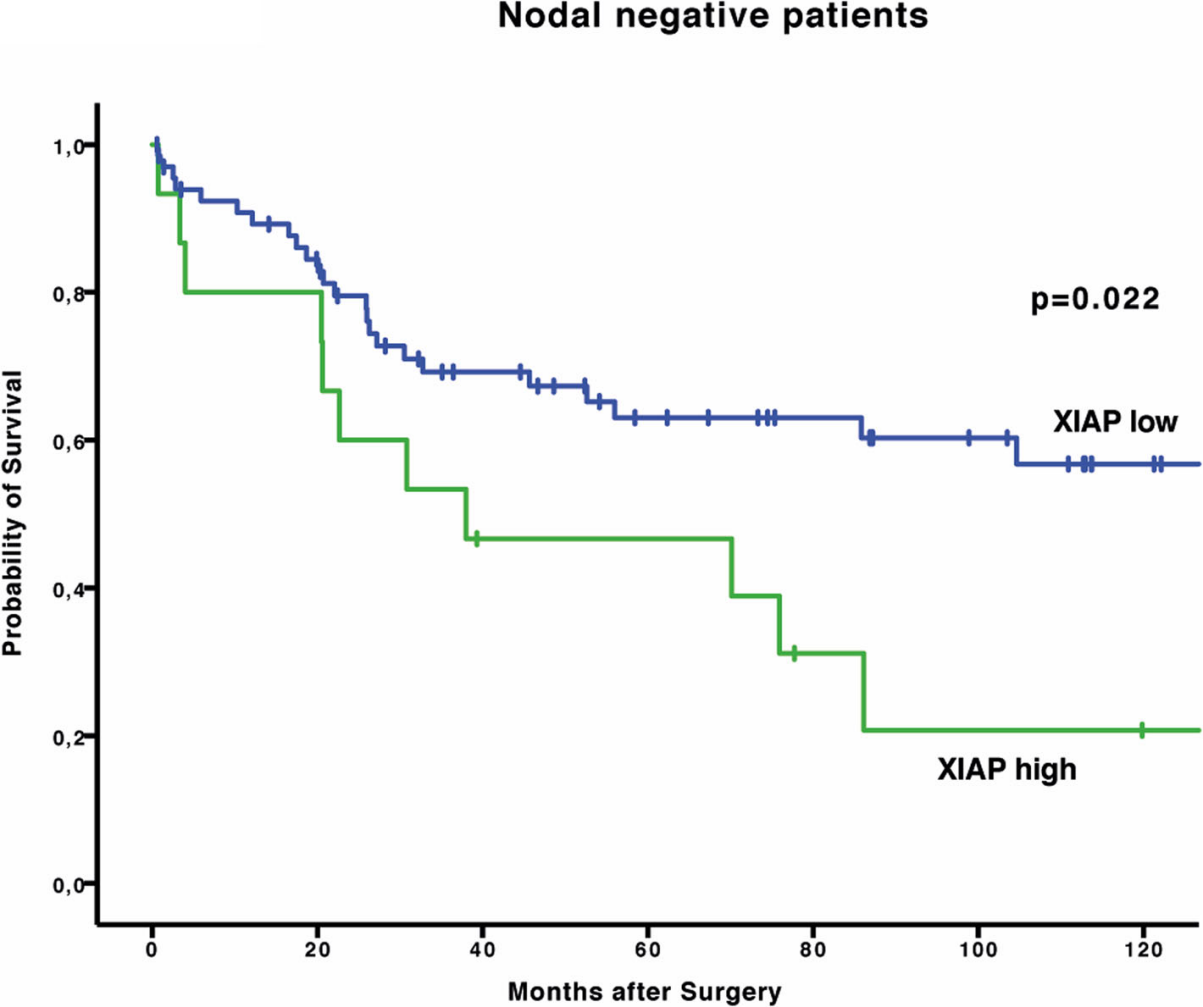
Kaplan-Meier curve showing OS of patients with nodal negative EAC stratified for XIAP low vs. high

On this basis we hypothesized that XIAP might be useful to define high risk situations in certain oncologic situations. We therefore analysed other specific subgroups that in general are believed to be favorable. First, we looked for nodal status. Patients that were found to be nodal negative had a significantly shortened survival when their intratumoral XIAP expression was high. OS in nodal negative, XIAP low patients was 202.2 months (95% CI 64.8–339.6 months, *p* = 0.546) while OS dropped drastically to 38.0 month in nodal negative, XIAP high patients (95% CI 0.00–94.8 months, *p* = 0.022), (Fig. 2).

Based on its capability to interfere with cellular death while promoting inflammatory signaling we hypothesized that XIAP might be particularly important in an immunoreactive tumor environment which is characterized by tumor infiltrating T-cells which execute a tumoricidal immune response under certain conditions. We stratified the patient collective for CD3 low and CD3 high tumors as CD3 positive T-cell infiltration is considered to be another setting of superior overall outcome [22].

**Fig. 3:**
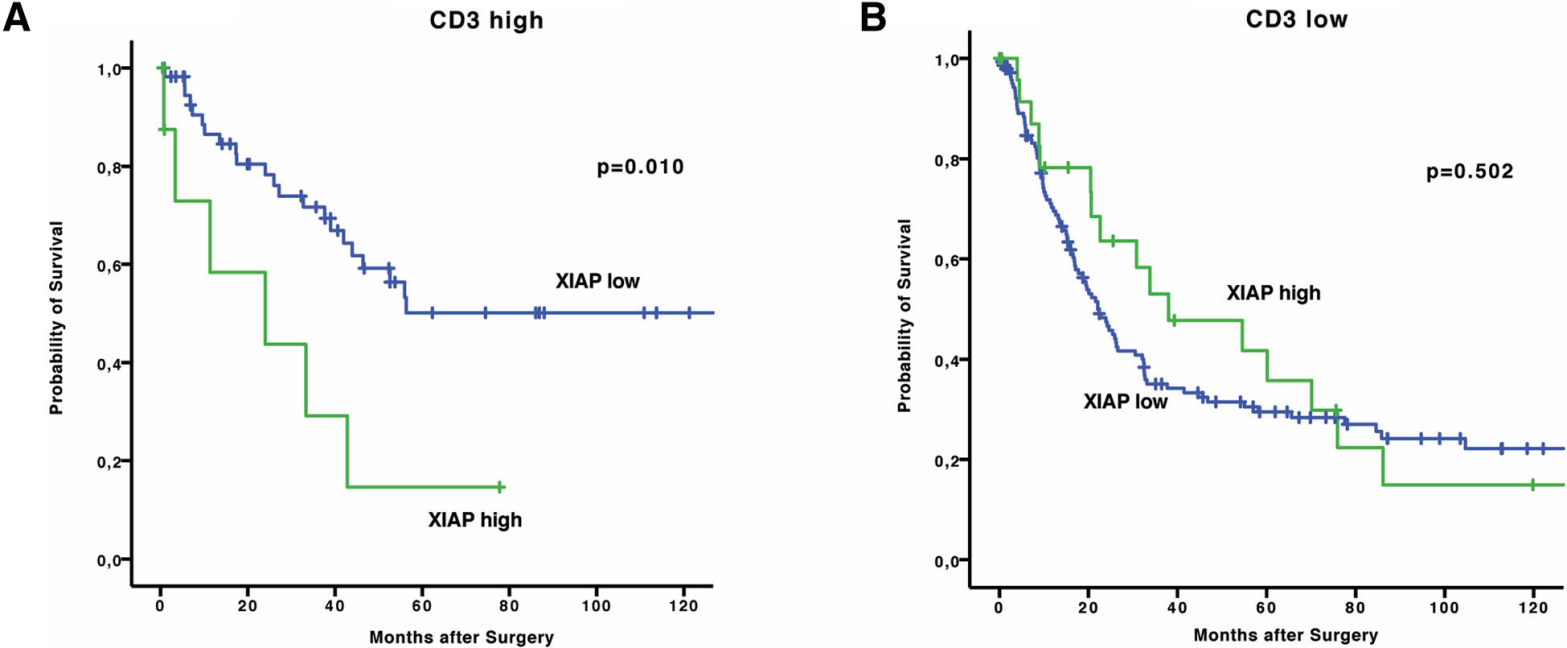
Survival curves depicting OS of patients with high (**a**) or low (**b**) T-cell infiltrated tumors (XIAP low vs. high tumors)

We found that in highly T-cell infiltrated tumors (CD3 high) high XIAP expression is detrimental for overall survival. Patients in this subgroup with low XIAP scores had a mean OS of 116.1 months (95% CI 89.6–142.6 months) whereas in patients with XIAP high tumors mean OS was 28.2 months (95% CI 9.7–46.6 months, *p* = 0.010), (Fig. 3a). This correlation was not apparent in CD3 low tumors (median OS 63.3 months (48.9–77.7) vs. 54.0 months (34.1–73.9), *p* = 0.502) (Fig. 3b).

To further verify our findings, we conducted multivariate analyses. In a cox-regression model of the whole study cohort, nodal status and grading were independent prognostic factors, XIAP was not associated with survival as expected. Within the CD3 high subgroup grading and XIAP (instead of nodal status) were independent prognostic factors for overall survival proving the significant role of XIAP in this subgroup (Table 3).

**Table 3:** Multivariate analyses of the whole study cohort and the CD3 high subgroup

			95% confidence interval		
		HR	lower	upper	*p*-value
study cohort	sex	1.673	0.773	3.623	0.192
	age	1.174	0.797	1.728	0.416
	nodal status	2.900	1.886	4.458	< 0.001
	grading	1.843	1.279	2.657	0.001
	XIAP	1.223	0.686	2.181	0.494
CD3 high subgroup	sex	0.598	0.102	3.501	0.568
	age	0.595	0.205	1.731	0.341
	nodal status	1.678	0.663	4.248	0.275
	grading	3.792	1.193	12.056	0.024
	XIAP	8.367	1.589	44.072	0.012

Our findings suggest that the tumor microenvironment is crucially involved in the effect of tumor cell XIAP on patient’s outcome. To strengthen this point we stratified for tumor cell genetic markers such as TP53, ERBB2 and ARID1A/SWI/SNF which would disregard corresponding tumor cell endogenous signaling pathways as the main driver of these effects. In fact, within any of these subgroups XIAP did not show an impact on overall survival (Additional file 1: Figure S1, Additional file 2: Figure S2, Additional file 3: Figure S3).

## Discussion

In the present study, we analyzed the protein expression of XIAP in a large and well characterized collective of esophageal adenocarcinomas. So, it was possible to perform subgroup analyses that could show the influence of XIAP on overall survival in specific patient groups. We could identify high levels of XIAP expression in malignant cells as a predictor of an immense negative prognosis in patients with nodal negative EAC who are at large considered to have a favorable outcome. We could furthermore show that elevated XIAP expression compromises overall survival in patients with highly T-cell infiltrated tumors that normally have a promising prognosis [22] and that patients that respond to chemotherapy and have high XIAP protein levels are at high risk.

Other studies previously reported a survival benefit for patients with lower expression of XIAP in comparison with patients expressing high levels of XIAP in esophageal squamous cell carcinoma (ESCC) but were not able to reveal a difference in OS in esophageal adenocarcinoma [23]. In contrast to the current study, only a relatively small group of patients was examined in which, due to the limited number of patients, sufficient subgroup analyses may not lead to significant results.

There are several possible scenarios that could explain our observation on a molecular level. Tumor cells with pathologically elevated XIAP levels are known to be more resistant to radio- or chemotherapy by reducing the capability to undergo therapy induced cell death [5]; it was already shown that reducing XIAP levels can re-sensitize cells to therapy induced apoptosis [24]. Since in our study collective, high XIAP expression was not correlated to a decrease in response to neoadjuvant therapy the observed effect cannot be explained by XIAP promoted therapy resistance only. In fact, we could observe that XIAP was therapy relevant in patients that are nodal negative, highly infiltrated by CD3 positive T-cells and had responded to therapy. It is therefore reasonable to think that the underlying mechanism is more complex and involves the tumor microenvironment rather than it is simply resistance to cell death that determines patients’ outcome, which is strengthened by the fact that expression/mutational status of conventional genetic markers did not influence XIAPs role in predicting overall survival.

Hypothetically cancer cell XIAP is involved in an immunomodulatory process that mitigates T-cell antitumor activity as XIAP is described to be involved in NFκB signaling [25, 26]. XIAP overexpression could result in constitutively activated NFκB leading to increased cytokine secretion that e.g. can recruit immune cells suppressing T-cell response or directly impede T-cell cytotoxicity [27]. Whether lymphangiogenesis as one prerequisite of nodal metastasis is involved in XIAP-related mechanisms and how immune cell infiltration besides T-cells is altered in XIAP low vs. XIAP high tumors is among the questions to be answered in the future. Anyhow, recently developed novel drugs that selectively target the pro-inflammatory properties of XIAP may provide an innovative therapeutic strategy in patients with EAC [26, 28].

## Conclusions

According to our data, XIAP could serve to identify high-risk patients within clinical low-risk groups according to the TNM staging system used so far. Those patients could be offered more aggressive therapy options (e.g. in XIAP high in nodal negative patients) or undergo extended surveillance after therapy. Since the current study is of retrospective character, future work should focus on prospective studies examining the impact of XIAP expression in specific patient subgroups. Additionally, pharmacological XIAP antagonization should be further evaluated in preclinical studies to evaluate its potential as targeted cancer therapy.

## Additional files

Additional file 1: **Figure S1.** Kaplan-Meier curve showing OS of patients with either wildtype TP53 **(A)** or mutated TP53 **(B)** stratified for XIAP low vs. high. (A) XIAP low: median OS 32.7 months (95% CI 17.9–47.6 months) vs. XIAP high: 33.3 months (95% CI 16.5–50.1 months). (B) XIAP low: median OS 30.5 months (95% CI 22.5–38.5 months) vs. XIAP high: 42.8 months (95% CI 23.3–62.3 months). (TIF 6349 kb)

Additional file 2: **Figure S2.** Kaplan-Meier curve showing OS of patients with either non-amplified erbb2 **(A)** or amplified erbb2 **(B)** stratified for XIAP low vs. high. (A) XIAP low: median OS 25.4 months (95% CI 19.1–31.7 months) vs. XIAP high: 30.8 months (95% CI 14.0–47.6 months). (B) XIAP low: median OS 55.0 months (95% CI 41.6–68.4 months) vs. XIAP high: 38.0 months (95% CI n.d.). (TIF 6349 kb)

Additional file 3: **Figure S3.** Kaplan-Meier curve showing OS of patients with either loss **(A)** or intact **(B)** arid1a expression (loss of expression describes scenarios of *ARID1a-Gen* alterations leading to a non-expressing protein - mainly related to mutation, deep deletion or promotor-methylation of *ARIDA1a*-gene or loss **(C)** or intact **(D)** SWI/SNF stratified for XIAP low vs. high. (A) XIAP low: median OS 22.1 months (95% CI 0–70.5 months) vs. XIAP high: 20.5 months (95% CI 0–45.0 months). (B) XIAP low: median OS 30.5 months (95% CI 24.3–36.7 months) vs. XIAP high: 38.0 months (95% CI 24.7–51.3 months). (C) XIAP low: median OS 30.5 months (95% CI 16.8–44.2 months) vs. XIAP high: 20.5 months (95% CI 0–42.8 months). (D) XIAP low: median OS 32.6 months (95% CI 23.2–42.0 months) vs. XIAP high: 42.8 months (95% CI 12.3–73.3 months). (TIF 13550 kb)

Additional file 4: **Figure S4.** Representative images of XIAP stained tumor sections of either XIAP low or high expressing tumors. Scale bar indicates 50 μm. (TIF 4549 kb)

## Abbreviations

EAC: Esophageal adenocarcinoma
ESCC: Esophageal squamous cell carcinoma
HR: Hazard ratio of death
TMA: Tissue-micro arrays
UICC: Union internationale contre le cancer
XIAP: X-linked inhibitor of apoptosis protein
