# Supplementary figures and images for: Elevated X-linked inhibitor of apoptosis protein (XIAP) expression uncovers detrimental prognosis in subgroups of neoadjuvant treated and T-cell rich esophageal adenocarcinoma
Source: BMC Cancer. 2019 May 31;19:531. doi: 10.1186/s12885-019-5722-1 (PMC6545033; doi:10.1186/s12885-019-5722-1)

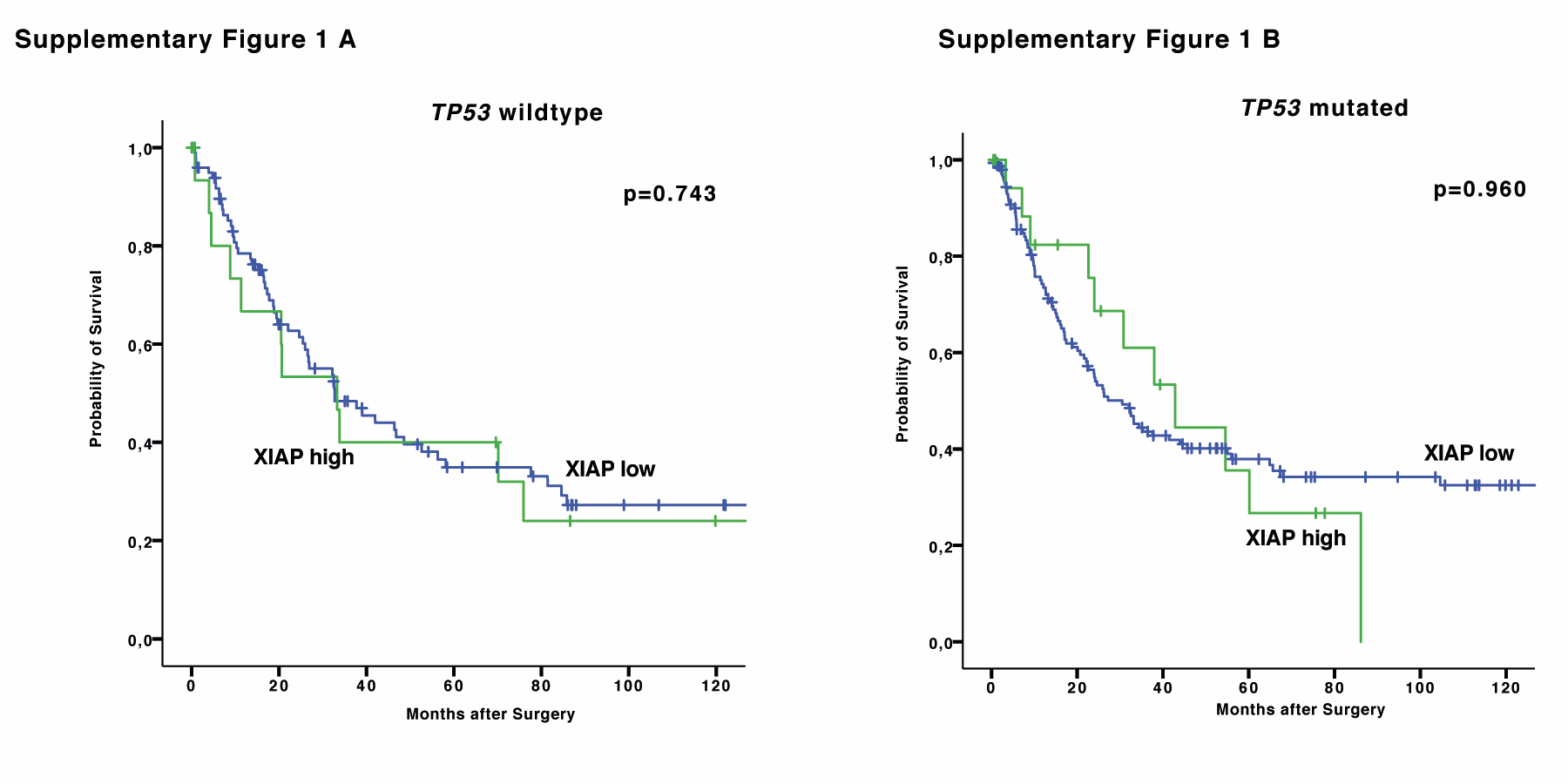

Supplement: Supplementary file 1 — Figure S1. Kaplan-Meier curve showing OS of patients with either wildtype TP53 (A) or mutated TP53 (B) stratified for XIAP low vs. high. (A) XIAP low: median OS 32.7 months (95% CI 17.9–47.6 months) vs. XIAP high: 33.3 months (95% CI 16.5–50.1 months). (B) XIAP low: median OS 30.5 months (95% CI 22.5–38.5 months) vs. XIAP high: 42.8 months (95% CI 23.3–62.3 months). (TIF 6349 kb) [file 12885_2019_5722_MOESM1_ESM.tif]

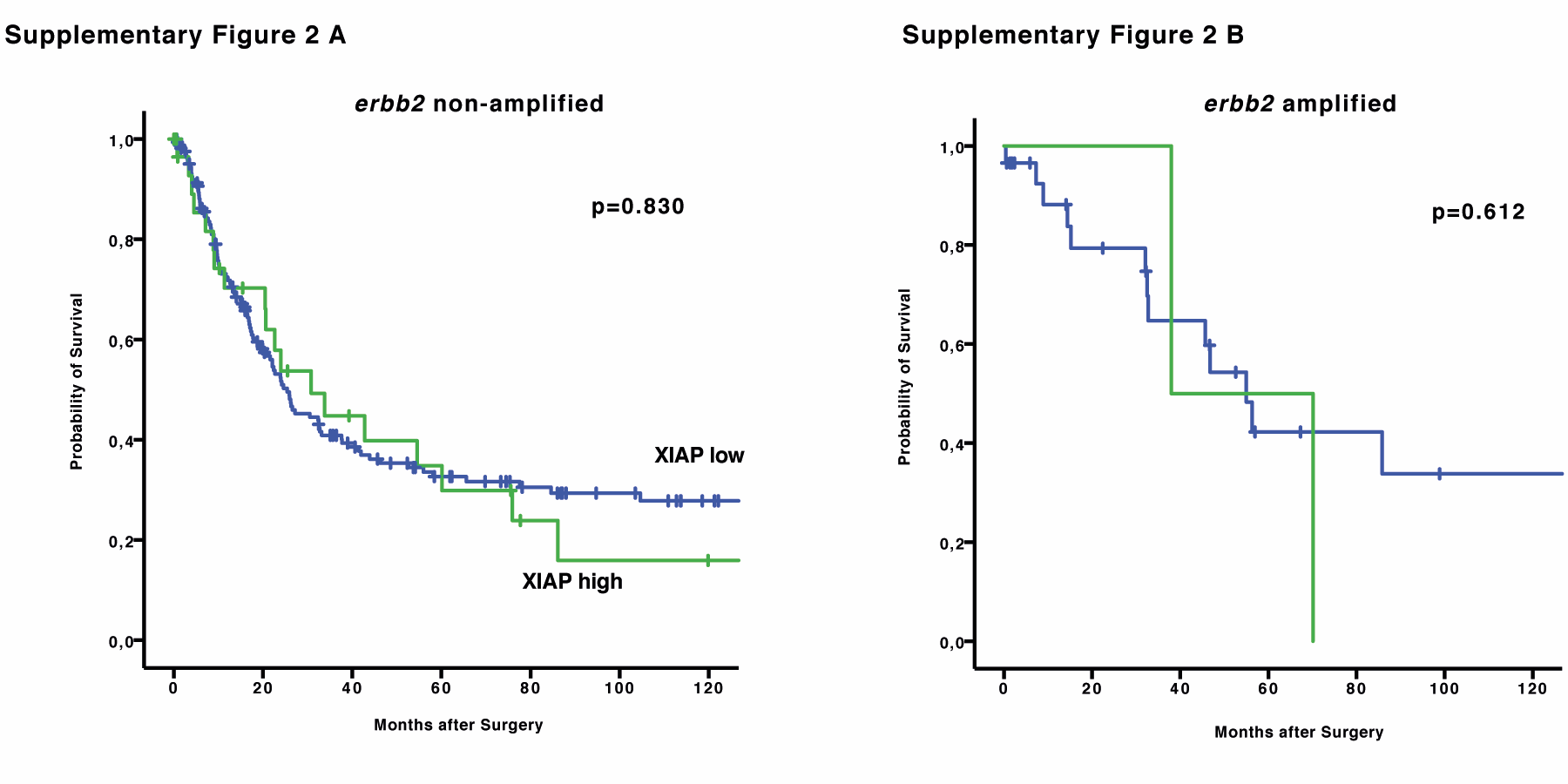

Supplement: Supplementary file 2 — Figure S2. Kaplan-Meier curve showing OS of patients with either non-amplified erbb2 (A) or amplified erbb2 (B) stratified for XIAP low vs. high. (A) XIAP low: median OS 25.4 months (95% CI 19.1–31.7 months) vs. XIAP high: 30.8 months (95% CI 14.0–47.6 months). (B) XIAP low: median OS 55.0 months (95% CI 41.6–68.4 months) vs. XIAP high: 38.0 months (95% CI n.d.). (TIF 6349 kb) [file 12885_2019_5722_MOESM2_ESM.tif]

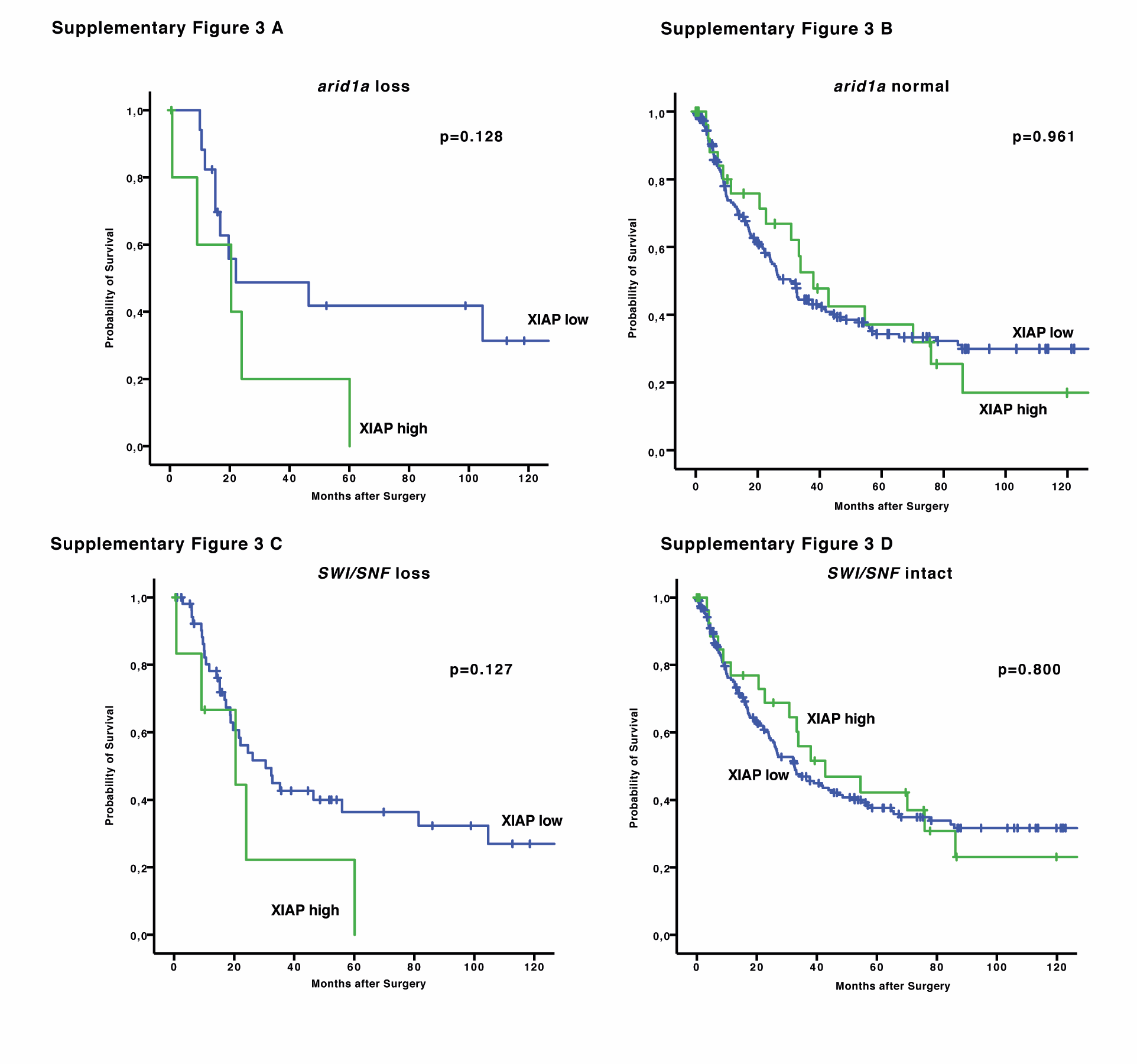

Supplement: Supplementary file 3 — Figure S3. Kaplan-Meier curve showing OS of patients with either loss (A) or intact (B) arid1a expression (loss of expression describes scenarios of ARID1a-Gen alterations leading to a non-expressing protein - mainly related to mutation, deep deletion or promotor-methylation of ARIDA1a-gene or loss (C) or intact (D) SWI/SNF stratified for XIAP low vs. high. (A) XIAP low: median OS 22.1 months (95% CI 0–70.5 months) vs. XIAP high: 20.5 months (95% CI 0–45.0 months). (B) XIAP low: median OS 30.5 months (95% CI 24.3–36.7 months) vs. XIAP high: 38.0 months (95% CI 24.7–51.3 months). (C) XIAP low: median OS 30.5 months (95% CI 16.8–44.2 months) vs. XIAP high: 20.5 months (95% CI 0–42.8 months). (D) XIAP low: median OS 32.6 months (95% CI 23.2–42.0 months) vs. XIAP high: 42.8 months (95% CI 12.3–73.3 months). (TIF 13550 kb) [file 12885_2019_5722_MOESM3_ESM.tif]

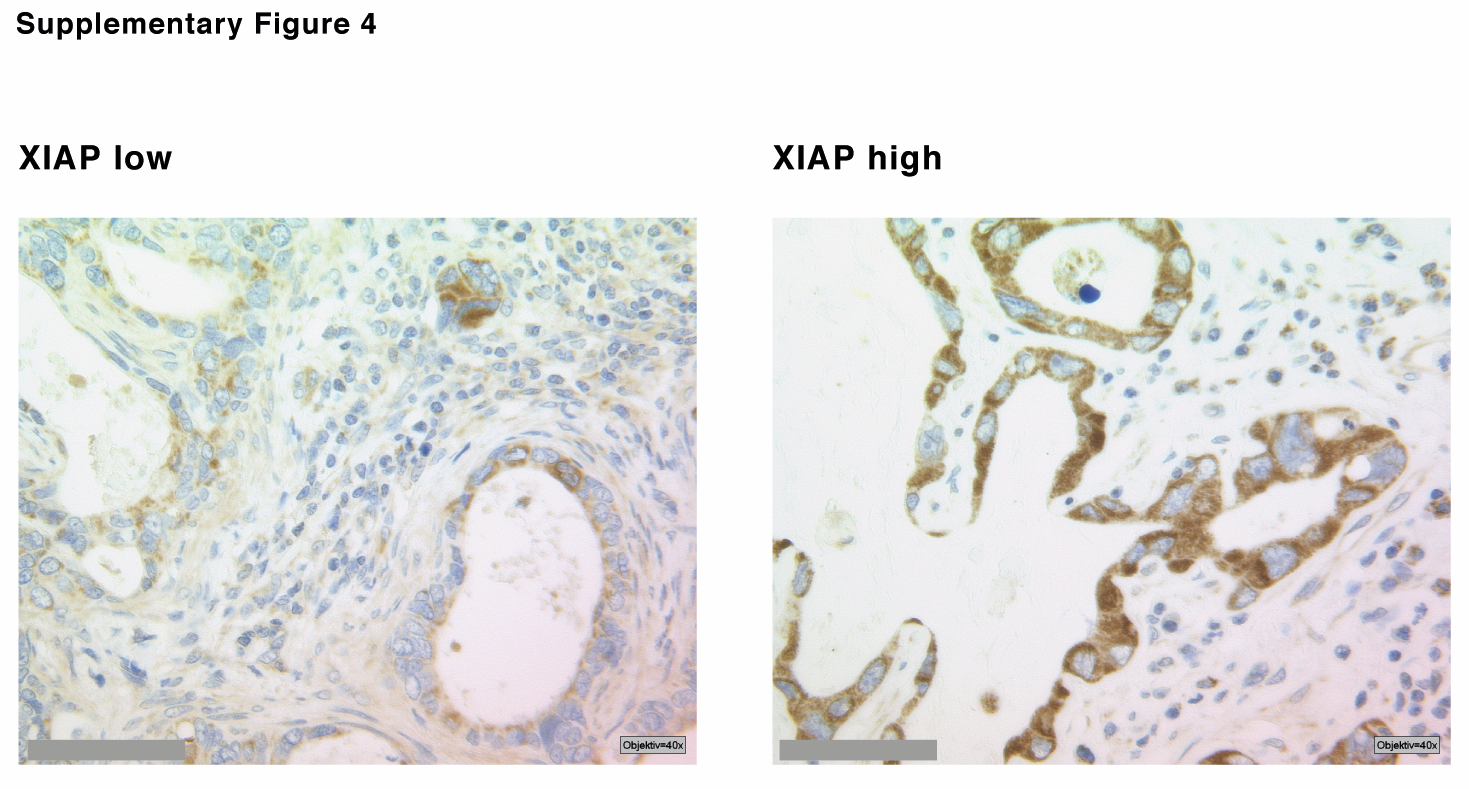

Supplement: Supplementary file 4 — Figure S4. Representative images of XIAP stained tumor sections of either XIAP low or high expressing tumors. Scale bar indicates 50 μm. (TIF 4549 kb) [file 12885_2019_5722_MOESM4_ESM.tif]
